# Age-treatment subgroup analyses in Cochrane intervention reviews: a meta-epidemiological study

**DOI:** 10.1186/s12916-019-1420-8

**Published:** 2019-10-21

**Authors:** Patrick Liu, John P. A. Ioannidis, Joseph S. Ross, Sanket S. Dhruva, Anita T. Luxkaranayagam, Vasilis Vasiliou, Joshua D. Wallach

**Affiliations:** 1 Yale School of Medicine, New Haven, CT 06510 USA; 2 Meta-Research Innovation Center at Stanford (METRICS), Stanford School of Medicine, Stanford, CA 94305 USA; 3 Department of Health Research and Policy, Stanford University School of Medicine, Stanford, CA 94305 USA; 4 Stanford Prevention Research Center, Department of Medicine, Stanford University School of Medicine, Stanford, CA 94305 USA; 5 Department of Statistics, Stanford University School of Humanities and Sciences, Stanford, CA 94305 USA; 6 Center for Outcomes Research and Evaluation (CORE), Yale-New Haven Health System, New Haven, CT 06510 USA; 7 Section of General Medicine, Department of Internal Medicine, Yale School of Medicine, New Haven, CT 06510 USA; 8 National Clinician Scholars Program, Yale School of Medicine, New Haven, CT 06510 USA; 9 Department of Health Policy and Management, Yale School of Public Health, New Haven, CT 06510 USA; 10 Department of Medicine, University of California, San Francisco School of Medicine, San Francisco, USA; 11 Section of Cardiology, San Francisco Veterans Affairs Health Care System, San Francisco, CA 94121 USA; 12 University of Connecticut, Storrs, CT 06269 USA; 13 Department of Environmental Health Sciences, Yale School of Public Health, New Haven, CT 06510 USA; 14 Collaboration for Research Integrity and Transparency (CRIT), Yale Law School, New Haven, CT 06510 USA

**Keywords:** Subgroup analyses, Precision medicine, Heterogeneity of treatment effects, Age subgroups

## Abstract

**Background:**

There is growing interest in evaluating differences in healthcare interventions across routinely collected demographic characteristics. However, individual subgroup analyses in randomized controlled trials are often not prespecified, adjusted for multiple testing, or conducted using the appropriate statistical test for interaction, and therefore frequently lack credibility. Meta-analyses can be used to examine the validity of potential subgroup differences by collating evidence across trials. Here, we characterize the conduct and clinical translation of age-treatment subgroup analyses in Cochrane reviews.

**Methods:**

For a random sample of 928 Cochrane intervention reviews of randomized trials, we determined how often subgroup analyses of age are reported, how often these analyses have a *P* < 0.05 from formal interaction testing, how frequently subgroup differences first observed in an individual trial are later corroborated by other trials in the same meta-analysis, and how often statistically significant results are included in commonly used clinical management resources (BMJ Best Practice, UpToDate, Cochrane Clinical Answers, Google Scholar, and Google search).

**Results:**

Among 928 Cochrane intervention reviews, 189 (20.4%) included plans to conduct age-treatment subgroup analyses. The vast majority (162 of 189, 85.7%) of the planned analyses were not conducted, commonly because of insufficient trial data. There were 22 reviews that conducted their planned age-treatment subgroup analyses, and another 3 reviews appeared to perform unplanned age-treatment subgroup analyses. These 25 (25 of 928, 2.7%) reviews conducted a total of 97 age-treatment subgroup analyses, of which 65 analyses (in 20 reviews) had non-overlapping subgroup levels. Among the 65 age-treatment subgroup analyses, 14 (21.5%) did not report any formal interaction testing. Seven (10.8%) reported *P* < 0.05 from formal age-treatment interaction testing; however, none of these seven analyses were in reviews that discussed the potential biological rationale or clinical significance of the subgroup findings or had results that were included in common clinical practice resources.

**Conclusion:**

Age-treatment subgroup analyses in Cochrane intervention reviews were frequently planned but rarely conducted, and implications of detected interactions were not discussed in the reviews or mentioned in common clinical resources. When subgroup analyses are performed, authors should report the findings, compare the results to previous studies, and outline any potential impact on clinical care.

## Background

Results from clinical trials support the actions of clinicians, patients, and policy-makers, but average treatment results may not apply to all patient subgroups [1]. Subgroup analyses attempt to refine interpretations about treatment effects (i.e., personalized or precision medicine) across various characteristics [2, 3]. Despite significant criticism about the validity of subgroup analyses [4–10], there is also mounting pressure and interest for various stakeholders, including regulators and research funders, to examine standard subgroups, such as age [11, 12]. The National Institutes of Health (NIH), the US Food and Drug Administration (FDA), and Cochrane encourages considering specific demographic subpopulations in the recruitment, analysis, and interpretation of trial results [13–19]. In theory, subgroup analyses should always be feasible to explore since information on age is routinely collected, and they may offer insights with relevance for clinical management [20, 21].

Meta-analyses can probe the validity of potential subgroup differences by collating evidence across multiple trials [22]. However, a previous analysis of 41 Cochrane reviews found that, despite interest in examining for possible treatment differences between males and females [13, 23, 24], only 7% of 109 sex-treatment subgroup differences were statistically significant, and they often had limited biological plausibility [25]. Little is known about age-treatment subgroup analyses in meta-analyses. Much like sex, age is a potentially important factor for decision-making [13, 26]. For instance, drug dosage or administration schedule for optimal treatment response will usually vary between children and older adults [27]. However, age subgroup analyses may also introduce additional analytical complexities, such as non-standardized age groups reported across trials [28, 29], which may pose obstacles to standardization in meta-analyses. Empirical evaluations using evidence on multiple topics and their meta-analyses have been conducted to compare results of pediatric and adult trials and have shown that there are sparse data to support investigations of heterogeneity between these age groups [30, 31], and there is limited evidence on subgroup differences being claimed for different adult age groups [32, 33].

In order to understand the conduct and clinical translation of age-treatment subgroup analyses, we used data from the Cochrane Database of Systematic Reviews to evaluate how often subgroup analyses of age are reported in Cochrane intervention reviews containing randomized trials, how often these subgroup analyses have statistical support (*P* < 0.05 from formal interaction testing), how frequently subgroup differences first observed in an individual trial are later corroborated by other trials in the same meta-analysis, and finally, how often statistically significant interactions are clinically relevant.

## Methods

### Study design and sample

We conducted a PubMed search using the Cochrane journal name tag (““Cochrane Database Syst Rev”[jour]”). The search retrieved 13,680 published articles indexed on PubMed on July 29, 2018 (date of download). We downloaded each article’s title, URL, authors, PubMed Identifier, and date of publication from PubMed. Considering that the PubMed search identified all Cochrane articles, including updates, Excel (version 14.7.6) was used to exclude duplicates (*n* = 4162) and withdrawn (*n* = 445) articles according to protocols defined by existing literature for retrieving and de-duplicating systematic reviews from PubMed [34]. When duplicates were identified, we selected the most recent version of the Cochrane review. Of the remaining 9073 studies, we used a random number generator to select 1000 articles for manual review (RStudio (version 1.1.42); Additional file 1: Table S1).

### Article and forest plot screening

One reviewer (PL) screened all 1000 articles to identify completed reviews of clinical interventions (i.e., “intervention reviews”) with plans to include randomized controlled trials. All protocols, diagnostic reviews, overviews, methodology reports, and editorials were excluded. We then reviewed the methods section for specification of any plans to conduct age-treatment subgroup analyses or any other statement that the authors conducted age-treatment subgroup analyses (Table 1). If intention was specified but subgroup analyses were not conducted, we noted if the reason why they were not conducted was stated (e.g., limited data available or no heterogeneity detected to warrant further analyses); if they were conducted, we recorded whether (and which) figure number(s) of forest plots were indicated within the text. Next, we screened all forest plots (title, footnotes, and plot contents) for any indication of age-treatment subgroup analyses. When reviews presented subgroup analyses across multiple forest plots rather than in a single forest plot, we matched and combined plots that were specific to a single age-treatment analysis (e.g., combining a forest plot for children and one for adults both with the same intervention [“lactulose versus polyethylene glycol”] and outcome [“relief of abdominal pain”]). A second reviewer (ATL) verified consistency and accuracy through a 5% random sample validation. The senior author (JDW) reviewed all age subgroup analyses, abstractions, and calculations.

**Table 1:** Subgroup analyses in Cochrane reviews

In meta-analyses, it is possible to formally test whether an intervention has different effects across subgroups based on patient, intervention, or study characteristics. When investigating subgroup differences in individual trials, subgroup analyses are based on within-trial comparisons. In other words, the subgroups of patients being compared come from the same study and population [35]. In meta-analyses, comparisons can be either within or between-trials. Within-trial comparisons are only possible if meta-analyses have access to individual participant level data and some of the trials contribute patients from every subgroup level considered [35]. However, subgroup analyses in meta-analyses are traditionally based on between-study comparisons, where certain trials only contribute data to one subgroup level [35]. When conducting a subgroup analyses for a meta-analyses, the two main steps are as follows: (1) calculating the effects within each subgroup level and (2) comparing the summary effects across the subgroup levels, using either fixed or random models within and between subgroups (e.g., a study can have random-effects within, and fixed effects between) [36]. In RevMan, the official Cochrane review software, a formal test for interaction is conducted using Cochran’s Q test, which tests the null hypothesis that the subgroup effects are the same and that any variation is no more than what would be expected by chance alone. The results of tests for interaction are typically present at the bottom of forest plots, with the text “Test for subgroup differences” [37]. If subgroup analyses are based on between-trial comparisons, the trials contributing data to different subgroup levels can have different patient, intervention, or study characteristics, which can complicate the conclusions from an interaction test [35].	

### Data abstraction

For all eligible age-treatment subgroup analyses identified in the reviews, one author (PL) abstracted the following characteristics: indication for the intervention, interventions compared, study population, subgroup levels (e.g., “adults,” “children”) and total number of levels, number of randomized controlled trials (total and for each individual subgroup level), sample size of individual trials (total and for each individual subgroup level), effect measure used in each analysis (e.g., risk ratio, odds ratio, rate ratio, mean difference, risk difference), method used for data synthesis (e.g., fixed effects or random effects), number of trials that contribute data to all subgroup levels presented in the analysis, and *P* value from a *χ*^2^ test for subgroup differences. We excluded age-treatment subgroup analyses that included any data from non-randomized controlled trials (i.e., quasi-randomized or observational). During this process, we also used the forest plots to note which subgroup analyses had overlapping (e.g., ages 2–12, 5–16, and 13–18) or non-overlapping (e.g., ages < 65 years and ages ≥ 65 years) age levels within the same forest plot. Two authors (PL and JDW) discussed all uncertainties, and an additional independent reviewer (JPAI) arbitrated all remaining discrepancies.

### Statistical analysis

Using descriptive statistics, we characterized the trials, study characteristics, and interventions of eligible Cochrane reviews. For all identified age-treatment subgroup analyses with non-overlapping age subgroup levels, we recreated the forest plots and then re-calculated interactions using the same methods outlined in the original Cochrane review (i.e., if the authors applied the Dersimonian and Laird random effects model to summarize risk ratios, we used the same effect measure and model). To determine whether standardization of the analyses affected the interpretation of the results, we re-evaluated the age-treatment interactions using (1) fixed and random effects (DerSimonian and Laird inverse-variance) models, and (2) risk ratio or mean difference effect measures, as previously described [25]. For each individual trial included in the subgroup analyses, we recorded the year of publication. For studies with no study year and/or with pre-pooling of multiple RCTs, we calculated the overall treatment interaction for the pooled data.

For each age-treatment subgroup analysis, we recorded whether a nominally statistically significant (*P* < 0.05) age-treatment interaction was seen in (1) the overall meta-analysis; (2) each individual trial contributing data for multiple subgroup levels (e.g., “above 50 years of age” and “below 50 years of age”); and (3) a meta-analyses containing only trials with data for all subgroup levels reported in the forest plot. For the analyses with a statistically significant treatment interaction, we then organized the trials by ascending year and noted whether the first (earliest) published trial contributing data for all subgroup levels had a significant treatment interaction. If so, we summarized the data from all other trials in the same subgroup analysis to determine whether the treatment interaction was statistically significant and whether the effect estimates in each of the subgroup levels were in the same direction (i.e., the subgroup analysis is corroborated). When evaluating corroboration of age-treatment interactions, we combined trials that had the same publication year in the same topic because the potential use of these trials as corroboration separately was unlikely.

### Analysis of clinical relevance of significant age-treatment interactions

For all age-treatment interactions that were explicitly reported in the forest plots by the review authors to be statistically significant and had non-overlapping age subgroups, we examined the full text of the Cochrane review to determine whether authors discussed biological plausibility or clinical relevance of the findings. For all eligible age-treatment interactions with statistically significant interaction *P* values, we conducted a search for suggestions of differential clinical management based on the age-treatment subgroup analyses in the following sources: BMJ Best Practice, UpToDate, Cochrane Clinical Answers, articles citing the Cochrane review (using Google Scholar), and a Google search of the first 10 pages for the intervention and type of subgroup (January 2019) (Additional file 1: Text 1).

### Sensitivity analyses

To minimize the possible correlation between analyses within the same Cochrane review, we used previously established criteria to determine two subsections of all subgroup analyses [25]. First, when reviews contained multiple subgroup analyses with identical or nearly identical outcomes for the same interventions, we selected only one analysis with non-overlapping subgroup levels according to the following algorithm: using the primary outcome described in the text, if available, and otherwise using the outcome with the most number of trials, or in the event of a tie, the smallest variance in the summary effect. If distinct outcomes for the same intervention were present, we noted all intervention-outcome analyses (i.e., at the forest plot level rather than at the review level). Second, we used the same criteria to select only one analysis per review.

We additionally examined statistical significance for age-treatment interactions based on a more stringent *P* value threshold for significance that has been recently proposed (*P* < 0.005) [38].

### Patient involvement

No patients were involved in setting the research question or the outcome measures, nor were they involved in developing plans for design or implementation of the study. No patients were asked to advise on interpretation or writing up of results. There are no plans to disseminate the results of the research to study participants or the relevant patient community.

## Results

### Search results

**Fig. 1:**
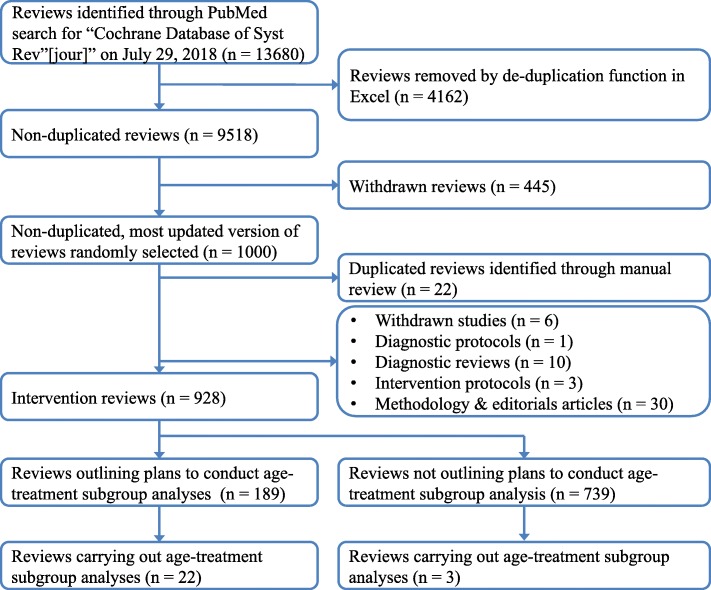
Flow diagram for inclusion and exclusion of Cochrane Reviews for age-treatment interactions

Among the 1000 randomly selected Cochrane articles, 22 (22 of 1000, 2.2%) were duplicate publications (i.e., Cochrane review updates) not identified by the automated de-duplication process and 6 (0.6%) were withdrawn studies. After further excluding diagnostic protocols (1, 0.1%), diagnostic reviews (10, 1.0%), intervention protocols (3, 0.3%), and overview, methodology, and editorial articles (30, 3.0%), a total of 928 intervention reviews (hereafter, “reviews”) remained (Fig. 1).

### Frequency of planned age-treatment subgroup analyses

Of the 928 reviews, 189 (20.4%) outlined plans to conduct age-treatment subgroup analyses in their methods sections (e.g., “we planned to investigate interactions by conducting subgroup analyses based on the following characteristics: age group of participants”). The vast majority of planned analyses (162 of 189, 85.7%) were either not conducted, or at least were not formally reported in forest plots. Common reasons for not conducting the 162 analyses were as follows: no studies were found for inclusion in the review (16, 9.9%); eligible studies were identified, but meta-analyses were not conducted (22, 13.6%); and eligible studies were identified, but there were insufficient data to conduct age-treatment analyses (71, 43.6%) (Additional file 1: Table S2). An additional 20 (12.4%) reviews did not provide a clear reason for not conducting/reporting the proposed subgroup analyses. After excluding five (2.6%) reviews that included subgroup analyses with non-randomized controlled trials, there were 22 (22 of 189, 11.6%; 22 of 928, 2.4%) that performed at least one age-treatment analysis containing only randomized controlled trials. Of the 739 reviews that did not explicitly include plans to conduct age-treatment subgroup analyses, three (0.4%) conducted and reported such analyses (Fig. 1).

### Characteristics of eligible Cochrane reviews

Of the 25 reviews that conducted at least one age-treatment subgroup analysis containing only randomized controlled trials (97 individual analyses, Additional file 1: Table S3), the publication years of the most updated version ranged from 2001 to 2018. The most common indications among the 97 individual analyses were respiratory (40 of 97, 41.2%), cardiovascular (19, 19.6%), and infectious (12, 12.4%) disease. There were 20 (20 of 25, 80.0%) reviews that had at least one analysis with non-overlapping age subgroup levels; four of these reviews also contained at least one subgroup analysis with potentially overlapping subgroup levels (e.g., age not categorized vs. ages < 2 years vs. ages 2–12 years). The remaining five reviews (5 of 25, 20.0%; 5 of 928, 0.5%) contained only subgroup analyses with potentially overlapping subgroup levels (Table 2). Additional details regarding study characteristics can be found in Additional file 1: Table S3.

**Table 2:** Age-treatment subgroup analyses with overlapping subgroup levels

There were 9 reviews with 32 individual age-treatment subgroup analyses based on potentially overlapping subgroup levels (e.g., mean age < 50 years, mean age 50 to < 65 years, vs. mean age 65+ years). The majority (25 of 32, 78.1%) of these subgroup analyses were in reviews that specified plans to conduct subgroup analyses in their methods section. Almost two thirds (20 of 32, 62.5%) of the analyses reported a *P* value from an interaction test, of which four (4 of 20, 20.0%) were statistically significant. Standardization by effect measures (mean difference or risk ratio) and a random effects model did not change the number of statistically significant age-treatment interactions; however, using a fixed effects model resulted in three additional statistically significant age-treatment interactions. Among the 12 age-treatment subgroup analyses without a reported *P* value from an interaction test, five analyses were from three reviews that outlined plans to conduct an age-treatment subgroup interaction test.	

### Age-treatment subgroup analyses in Cochrane reviews

The 20 reviews with at least one age-treatment subgroup analysis with non-overlapping subgroup levels conducted a total of 65 individual analyses with non-overlapping subgroup levels, with a median of two (interquartile range [IQR], 1–4) analyses per review. Of these 65 analyses, five (7.7%) included only pediatric populations and 14 (21.5%) included only adult populations. There were 46 (46 of 65, 70.8%) that included both pediatric and adult populations, of which 37 (37 of 46, 80.4%) had “children” and “adults” as the two subgroup levels. The 65 analyses contained a total of 184 unique randomized controlled trials. The median number of trials with age subgroup data per analysis was three (IQR, 2–8.5), and the median sample size among the 61 analyses reporting samples sizes was 810 (IQR, 355–2545). Approximately one third (24 of 65, 36.9%) of the analyses contained only one trial per subgroup level.

The most common effect measures were the mean difference (40 of 65, 61.5%) and risk ratio (14 of 65, 21.5%) (Additional file 1: Table S4). All analyses except one used inverse-variance (46 of 65, 70.8%) or Mantel-Haenszel (18 of 65, 27.7%) methods. Fixed rather than random effects models were commonly used (52 of 65, 80.0%).

### Frequency and characteristics of statistically significant age-treatment interactions

Among the 65 analyses with non-overlapping subgroup levels, 51 (78.5%) reported a *P* value from an interaction test, of which seven (7 of 53, 13.7%) were statistically significant at *P* < 0.05 (Table 3, Additional file 1: Table S5). The results of standardization using fixed and random effects models are available in Additional file 1: Text 2.

**Table 3:** Summary results for proportion of statistically significant age-treatment interactions among subgroup analyses with non-overlapping subgroup levels

	No. (%) of statistically significant age-treatment interactions		
	*P* value for interaction reported in forest plots	*P* value for interaction not reported in the forest plots	Total
All age-treatment analyses			
Using analytical methods reported in forest plots^a^	7/51 (13.7)	4/14 (28.6)	11/65 (16.9)
Standardized using a fixed effects model^b^	8/49 (16.3)	5/14 (35.7)	13/63 (20.6)
Standardized using a random effects model^b^	7/49 (14.3)	4/14 (28.6)	11/63 (17.5)

^a^We recreated the forest plots using the same methods outlined in the original Cochrane review (i.e., if the authors applied the Dersimonian and Laird random effects model to summarize risk ratios, we use the same effect measure and model)

^b^When standardizing using fixed and random effects models, we excluded two subgroup analyses from one Cochrane review that did not provide information on which studies were included in the subgroup analyses or the methodology for the subgroup analyses that they conducted

When limited to the 16 analyses (16 of 65, 24.6%) with at least one trial contributing data for all subgroup levels in an analysis, only one (1 of 16, 6.3%) age-treatment interaction was statistically significant (using either fixed or random effects models). There were four (4 of 16, 25.0%) analyses with multiple trials and at least one trial contributing data for all subgroup levels, of which none (0 of 4, 0.0%) had a statistically significant age-treatment interaction. Two age-treatment analyses from the same review were not considered as including trials contributing data to all subgroup levels because the authors did not specify the individual trials used to perform the analyses.

### Corroboration of significant age-treatment subgroup analyses

Among the four age-treatment subgroup analyses that included multiple trials with at least one trial contributing data for all subgroup levels, seven individual trials with data for all subgroup levels could be tested for an age-treatment interaction. Only one (14.3%) of these seven trials had a statistically significant age-treatment interaction, and it was the first (earliest) published trial included in the analysis. For the analysis including that one trial, there was only one other trial included in the analysis, and it did not corroborate the statistically significant interaction.

### Clinical translation of statistically significant age-treatment interactions

Of the seven age-treatment subgroup analyses that reported a statistically significant *P* value from an interaction test, two were from reviews with the same authors [39, 40]; otherwise, there was not a clear pattern of interventions, comparisons, and outcomes (Table 4). Six (6 of 7, 85.7%) were from reviews that outlined plans to conduct subgroup analyses in their methods sections. Five (5 of 7, 71.4%) had treatment effects in the same direction for all age subgroup levels. None of the seven analyses (0 of 7, 0.0%) had at least one trial contributing data for all subgroup levels. Furthermore, none (0 of 7, 0.0%) of the reviews containing statistically significant age-treatment interactions discussed biological rationale or clinical relevance in their Discussion or Implications for Practice sections. For all seven statistically significant age-treatment interactions, there was no discussion of the age-treatment differences on BMJ Best Practice, on UpToDate, on Cochrane Clinical Answers, in articles citing the Cochrane review (using Google Scholar), and on Google.

**Table 4:** Characteristics of seven statistically significant age-treatment interactions reported by authors

Comparison	Population characteristics	Outcome	Primary outcome^*^	Specified in methods?^†^	Reported age subgroup levels	Effect size [95% CI]^‡^	RCTs per subgroup level	RCTs across all levels^§^	*P* value	Biological/clinical rationale^¶^
Botulinum toxin vs surgery (2017)	Children and adults with strabismus suitable for treatment with botulinum toxin to align the angle of deviation	Improved ocular alignment ≤ 10 prism dioptres	Yes	No	Children	RR 0.91 [0.71, 1.16]	2	0	0.04	No
					Adults	RR 0.38 [0.17, 0.85]	1			
Digital intervention vs no/minimal intervention (2017)	Individuals in the community with hazardous or harmful alcohol consumptions directed toward any digital intervention	Quantity of drinking (grams/week), based on the longest follow-up	Yes	Yes	Adolescents/young adults	MD − 13.44 [− 19.27, − 7.61]	28	0	0.002	No
					Adults	MD − 56.05 [− 82.08, − 30.02]	14			
Supplementary feeding vs comparator by age of children (2012)	Children from low- and middle-income countries born at term, from birth to 5 years old	Weight gain (kg) during the intervention	Yes	Yes	Children younger than 24 months	MD − 0.01 [− 0.09, 0.07]	4	0	0.008	No
					Children older than 24 months	MD 0.22 [0.07, 0.37]	1			
Diet plus physical activity vs comparator (2017)	Individuals diagnosed with intermediate hyperglycemia or prediabetes at increased risk of developing type II diabetes mellitus	Incidence of type 2 diabetes	Yes	Yes	Ages < 50 years	RR 0.70 [0.57, 0.85]	2	0	0.009	No
					Ages ≥ 50 years	RR 0.50 [0.44, 0.58]	9			
		2-h plasma glucose	No	Yes	Ages < 50 years	MD − 1.62 [− 2.49, − 0.76]	2	0	0.003	No
					Ages ≥ 50 years	MD − 0.27 [− 0.49, − 0.05]	7			
Colony-stimulating factor plus antibiotics vs antibiotics alone (2014)	Individuals undergoing chemotherapy for cancer who experienced neutropenia and fever	Time to neutrophil recovery	No	Yes	Children	RR 0.80 [0.66, 0.97]	1	0	0.02	No
					Adults	RR 0.45 [0.29, 0.70]	5			
Fluticasone propionate vs beclomethasone dipropionate or budesonide, parallel-group studies: dose ratio 1:2 subgroup by age (2007)	Children (> 2 years) and adults with a clinical diagnosis of asthma	Change in Forced Expiratory Volume 1 compared to baseline	Unclear	Yes	Children	MD − 0.04 [− 0.10, 0.02]	2	0	0.02	No
					Adults	MD 0.04 [0.00 0.08]	10			

*MD* mean difference, *RCT* randomized controlled trial, *RR* risk ratio

^*^Is the outcome for this analysis specified as the primary outcome in the text of the review?

^†^Is the age-treatment subgroup analysis outlined in the Methods section of the review?

^‡^Effect sizes are reported using the statistical methods specified in the forest plot

^§^Subgroups with RCTs that include data for all subgroup levels of the analysis

^¶^Did the reviews discuss the subgroup analyses and provided clinical or biological rationale?

### Sensitivity analysis

After selecting the subset of age-treatment subgroup analysis with unique outcomes for the same comparison, 38 (58.5%) of the 65 analyses with non-overlapping subgroup levels remained (Additional file 1: Table S5). Nine (9 of 38, 23.7%) analyses had a statistically significant age-treatment interaction. When one analysis was chosen for each of the 20 reviews with non-overlapping subgroup levels, six (6 of 20, 30.0%) had a statistically significant age-treatment interaction. The results of standardization using fixed and random effects models are available in Additional file 1: Text 2.

Only two (28.6%) of the seven age-treatment subgroup analyses that reported a statistically significant *P* value from an interaction test at the 0.05 level were still significant after lowering the threshold to 0.005 (2 of 7, 28.6%; 2 of 51 analyses that reported a *P* value, 3.9%). Among the four statistically significant interactions from analyses reported in forests plots without a *P* value from an interaction test, one was significant using a *P* value threshold less than 0.005 (1 of 4, 25.0%; 1 of 14 analyses that did not report a *P* value; 7.1%) (Additional file 1: Table S5).

## Discussion

Among 928 randomly selected Cochrane intervention reviews, one fifth outlined plans to conduct age-treatment subgroup analyses. However, only 25 (2.7%) reviews actually reported age-treatment subgroup analyses containing only randomized controlled trials in their forest plots. Of these, there were 20 reviews, with 65 separate analyses, that used non-overlapping age subgroup levels. While seven analyses (10.8%) reported a statistically significant *P* value from an interaction test, none of the corresponding reviews provided biological rationale or discussed the clinical relevance of their statistically significant subgroup differences. Additionally, none were subsequently summarized in commonly used resources for clinical management.

While Cochrane intervention reviews often planned age-treatment subgroup analyses, few of them were conducted, and lack of available age-related data was a common obstacle. A recent evaluation of 116 Cochrane HIV systematic reviews also found that 21 of 49 (42.9%) reviews with no meta-analyses cited insufficient studies/data for the inability to conduct subgroup analyses [41]; age was the second most commonly planned factor for subgroup analyses but not among the top five most frequent factors actually used in subgroup analyses [41]. Prior research on Cochrane pediatric meta-analyses observed that just over one fifth of pediatric reviews published in 2011 performed age-treatment subgroup analyses [29]. Together, these findings suggest that, despite being viewed as important sources of evidence for patients and clinicians, reviews are unlikely to derive personalized treatment effect estimates related to age in subgroup analyses [10, 42–44].

The age-treatment subgroup analyses in Cochrane intervention reviews often included overlapping groups (e.g., children vs. adults vs. unclear). This is likely because individual trial publications provide only limited summary-level information [13, 19, 45–47]. While previous studies have outlined concerns about the conduct and interpretation of subgroup analyses in individual clinical trials [48–50] and sex-treatment subgroup analyses in Cochrane reviews [25], our findings show that these problems also extend to the conduct of age subgroup analyses at the review level [5, 9, 48, 51].

Among the age subgroup analyses with non-overlapping subgroup levels, the prevalence of statistically significant *P* values reported by authors was higher than what would be expected by chance (5%). However, there was no corroboration of statistically significant age-treatment interactions from individual trials by other trials within each of the 12 meta-analyses. We observed a higher proportion of statistically significant age-treatment interactions when only one analysis was selected per Cochrane review (40.0% when standardized to using a fixed effects model). Nevertheless, many statistically significant findings had subgroup level effect estimates in the same direction, were based on a small number of trials, and are likely spurious. Therefore, it may not be surprising that none of the Cochrane intervention reviews that reported statistically significant findings also discussed the results from the age subgroup analyses. These findings are consistent with a previous evaluation of sex-treatment subgroup analyses in Cochrane reviews, where statistically significant interactions were rare (7%), often included only one trial, and lacked corroboration [25].

The majority of the non-overlapping subgroup analyses that we found pertained to differences between adults and children. A previous empirical evaluation assessed differences in effectiveness of treatments for adults and children and concluded that there is often limited evidence to arbitrate if heterogeneity exists in these two age populations [31]. Given that adult data are often extrapolated to children, where evidence is often scant [52], this lack of evidence is problematic. The same applies to other age comparisons, such as those involving adult populations of different ages, especially the older adults who are often underrepresented in trials [53].

### Limitations

Our evaluation is based on a relatively small number of age-treatment subgroup analyses. However, we obtained these from a large random sample of all Cochrane reviews; therefore, our findings are expected to be generalizable to Cochrane reviews broadly. Although age-specific effects may be presented in separate reviews (e.g., one on children and one on adults, or in reviews focused on the elderly), we did not collate these reviews, since evaluating age-treatment interactions from data presented in different reviews would pose methodological challenges. We cannot determine how many subgroup analyses were performed but not reported (e.g., because of non-significant results or because authors felt that the data were too limited). We did not screen all of the Cochrane protocols for planned age-treatment subgroup analyses that were not reported or conducted in the Cochrane reviews, and subgroup analyses may be added after the protocol. However, according to the Methodological Expectations of Cochrane Intervention Reviews (MECIR) manual, which provides “standards for conducting and reporting of new Cochrane Intervention reviews, reporting of protocols and the planning, conducting and reporting of updates,” when subgroup analyses are performed, authors should “explain and justify any changes from the protocol (including any post hoc decisions about eligibility criteria or the addition of subgroup analyses)” [54]. Accordingly, our focus was on what was reported in Cochrane reviews. Finally, three Cochrane reviews containing four age-treatment subgroup analyses with statistically significant results from an interaction test had updated versions published in 2017, which was within two years of our analysis of clinical relevance. Because translation of results of meta-analyses to clinical practice guidelines may take time, it is not known if these results were included in current clinical practice guidelines.

### Implications

Our findings demonstrate the dearth of insight gleaned from age subgroup analyses conducted to date. Current guidelines exist related to the analysis of clinical data by age subgroups. The FDA, citing low reporting and inconsistent evidence for analyzing treatment differences across age for medical devices, has released recommendations for age subgroup analyses [19, 26]. The Cochrane Equity Methods Group encourages authors to explore characteristics, including age, that can stratify health outcomes [17]. Cochrane also provides guides for authors regarding best practices for exploring heterogeneity among different participant factors [22]. Furthermore, the Cochrane Handbook outlines that “investigations of characteristics of studies that may be associated with heterogeneity should be pre-specified in the protocol of a review” and warns that “investigations of heterogeneity when there are very few studies are questionable in value” [22]. However, our study suggests a lack of awareness of or adherence to these guidelines and demonstrates the need for improved pre-specification of subgroup analyses in reviews, including adequate descriptions outlining potential biological and clinical rationale supporting the conduct of age-treatment analyses. If subgroup analyses cannot be performed, authors should discuss potential barriers preventing their analyses. When subgroup analyses are performed, authors should consider limitations (e.g., small number of trials), report all findings, compare the results to previous studies, and outline any potential impact on clinical care [48].

Retrieving age-related data across different randomized controlled trials may be hindered by lack of standardized age categories and poor reporting (e.g., trials reporting mean ages or providing unclear age information). Improved data standardization and reporting practices are necessary for individual trials. Stakeholders, particularly the Clinical Data Interchange Standards Consortium, should work together to establish greater consistency across trials that will allow for improved demographic subgroup reporting [13]. Moreover, increased access to and utilization of individual participant data (IPD) can help address the challenges associated with availability of data used to investigate subgroup differences [29, 55]. For meta-analyses that identify an adequate number of trials, cumulative subgroup analyses using IPD have been recommended for “individualized medicine” [56]. However, a survey of published IPD meta-analyses shows that only a minority find strong evidence for subgroup differences [55].

## Conclusions

In this evaluation of 928 randomly selected Cochrane interventions reviews, one-fifth outlined plans to conduct age-treatment subgroup analyses. However, the vast majority cited insufficient data as the reason for being unable to carry out their planned analyses, and less than 3% actually performed at least one age-treatment subgroup analysis. None of the reviews provided biological or clinical rationale for subgroup differences or had age-treatment results that were referenced in common resources for clinical management. Research intending to identify differential treatment effects between age groups can benefit from standardization at all levels, from demographic data reporting in individual trials to synthesis and reporting of subgroup analyses in meta-analyses.

## Supplementary information

**Additional file 1:** **Table S1.** PMIDs of screened articles, excluding duplicate articles. **Text 1.** (Search terms for evaluating evidence of statistically significant results included in clinical management guidelines for 7 statistically significant age-treatment subgroup analyses). **Table S2.** (Reasons for not performing age-treatment subgroup analyses among 162 Cochrane intervention reviews). **Table S3.** (Characteristics of 97 age-treatment subgroup analyses from 25 Cochrane intervention reviews). **Table S4.** (Meta-analytical methods used by authors in their age-treatment interactions). **Table S5.** (Summary results for proportion of statistically significant age-treatment interactions based on different characteristics and criteria among subgroup analyses with non-overlapping subgroup levels). **Text 2.** (Standardization using only fixed and only random effects models). (PDF 1491 kb)

## Data Availability

The datasets used and/or analyzed during the current study are available from the corresponding author on reasonable request (Joshua.wallach@yale.edu).

## Abbreviations

CI: Confidence interval
IQR: Interquartile range
FDA: Food and Drug Administration
NIH: National Institutes of Health
