# Supplementary material for: Age-treatment subgroup analyses in Cochrane intervention reviews: a meta-epidemiological study
Source: BMC Med. 2019 Oct 21;17:188. doi: 10.1186/s12916-019-1420-8 (PMC6805640; doi:10.1186/s12916-019-1420-8)
Supplement: Supplementary file 1 — Additional file 1: Table S1. PMIDs of screened articles, excluding duplicate articles. Text 1. (Search terms for evaluating evidence of statistically significant results included in clinical management guidelines for 7 statistically significant age-treatment subgroup analyses). Table S2. (Reasons for not performing age-treatment subgroup analyses among 162 Cochrane intervention reviews). Table S3. (Characteristics of 97 age-treatment subgroup analyses from 25 Cochrane intervention reviews). Table S4. (Meta-analytical methods used by authors in their age-treatment interactions). Table S5. (Summary results for proportion of statistically significant age-treatment interactions based on different characteristics and criteria among subgroup analyses with non-overlapping subgroup levels). Text 2. (Standardization using only fixed and only random effects models). (PDF 1491 kb) [file 12916_2019_1420_MOESM1_ESM.pdf]

Age-treatment subgroup analyses in Cochrane intervention reviews: a meta-epidemiological study

Patrick Liu; John PA Ioannidis; Joseph S Ross; Sanket S Dhruva; Anita T Luxkaranayagam; Vasilis Vasiliou; Joshua D Wallach

**Additional file 1: APPENDIX FILE 1**	**Page #**
**table S1:** PMIDs of screened articles, excluding duplicate articles	
**text 1.** Search terms for evaluating evidence of statistically significant results included in clinical management guidelines for 7 statistically significant age-treatment subgroup analyses	2
**table S2**: Reasons for not performing age-treatment subgroup analyses among 162 Cochrane intervention reviews	6
**table S3**. Characteristics of 97 age-treatment subgroup analyses from 25 Cochrane intervention reviews	9
**table S4**: Meta-analytical methods used by authors in their age-treatment interactions.	20
**table S5.** Summary results for proportion of statistically significant age-treatment interactions based on different characteristics and criteria among subgroup analyses with non-overlapping subgroup levels.	21
**text 2.** Standardization using only fixed and only random effects models.	23

table S1: PMIDs of screened articles, excluding duplicate articles									
23794201	29078005	27925149	26455722	25344083	24142844	26333656	19160244	16235288	23450535
23771729	29077194	27918616	26424726	25338726	24129886	21678381	19160216	16235370	12519573
23771694	29064090	27906452	26421585	25321859	24108523	21678341	29265171	19821315	26141572
23744563	29058762	27883192	26419832	22786510	24085592	21678358	18843641	16235377	19160181
23744560	29041034	27875631	26415762	25310725	24085633	30091808	18843687	27467116	20614422
23740540	29036756	27820878	26397173	25300594	24065550	21563133	18843663	16235395	29578611
23740790	23235662	27734465	26387658	25288182	24030739	21563129	24760679	28114727	10796438
23740535	23235610	27727432	26384035	25274134	24027084	21563171	18843711	19821275	10796346
23737086	23235590	27797129	26368505	25254615	24022428	21563135	22972086	16034932	10796794
23728688	23235617	27756113	26361135	25247266	24006231	21491400	18677782	25829028	10796212
23728637	23235630	27783843	26343254	25234029	23999923	21412885	26058964	16034956	25602133
23728679	23152245	27696349	26310586	25229700	23996298	27552162	18646136	16034939	10796519
23728650	23152280	30095853	26308931	25198378	23996271	21328304	18646114	16034887	10796458
23728684	23152216	27684492	26305459	25184502	23990391	23235654	25940444	16034926	10796775
23633365	29096046	27670126	26301526	25178118	23983042	28880995	19821311	30156270	10796861
23633303	23152236	27648846	26284429	25159027	23949842	21249676	18646155	15846606	11869564
23633380	23152230	27647489	26228086	25140869	23955506	21249701	18646107	24934383	10796784
23633368	23152242	27644167	26222245	25133355	23934958	21249700	18425884	27210414	23152214
23543535	23076942	27618521	26202854	25121931	23900725	21249703	24366859	15846622	23152274
23543515	23076913	27611234	26198593	25110856	23897513	21249714	18425938	15846643	22895949
23543527	23076963	27602537	26197477	25101365	23897547	21249684	18425886	25435250	22696357
23543532	23076886	27589694	26184396	25066627	23881649	21249664	18425930	27106604	22513951
23543542	23076892	27582266	26171903	25072817	23881659	21249674	18425975	15674961	22419277

23543586	28737834	27577848	26171908	25069620	23881650	21154360	18425928	15674916	22336820
23543545	23076903	27574798	26171901	25010869	23857562	21154372	29920657	15674968	22071851
23450547	22972094	27572944	26171899	24995856	23824785	21069679	18253983	15654705	21975786
30073665	22972082	27562656	26171897	24980859	23818069	21069685	18254069	15495040	17636804
23450559	22972088	27552522	26147958	24957503	23813393	21069686	24166716	15495071	17443616
23450534	22972150	27535773	26147896	24953826	23813336	20927755	18254085	22592695	17443568
23450578	22972115	27502170	26133923	24937099	23813425	20927748	18646161	19160232	17253491
23450580	22895933	27465317	26133313	24924489	23799857	20927747	18254089	15495123	17054134
23440799	28984368	27457661	26134172	24915581	23797676	20927774	19370610	15495067	17054252
23440818	28965364	27450741	26133124	24896368	29893410	20927758	17943920	15494987	17054130
23440810	28921500	27428009	26130018	24880031	26731032	29870574	17943795	15495128	16855984
23440784	28905362	27409709	26123284	24872328	26661390	20824876	17943806	15266530	16235301
23440794	28898400	27405706	26106870	24859467	26621223	20824844	29926476	15266472	22513913
29489029	28895125	27387849	26089258	24848732	26184394	20687097	26830050	15266524	15846645
23440829	28891235	27384151	26079202	24832784	25754617	20687091	22972055	16625539	14973967
23440816	28891588	27357126	26068707	30101972	25730178	20687062	17943807	24338792	25564770
23440830	28881007	27314455	26041152	22786522	25702785	20556753	17636750	15266427	25188769
23440813	28854319	30113083	25994229	22696365	29063594	20556756	17636735	30039853	10908531
23440805	28832903	27195724	25991068	22696387	24353242	20556757	17636702	14973994	10796605
23235651	28832911	27157143	25985145	22696371	24173771	20464725	17636780	14974046	26517527
23235635	28779487	27149547	25966446	22592678	23963712	20464741	17636726	14973991	20927720
26597166	28770973	27142842	25961741	24823491	22592725	20464736	17636665	14973992	24913720
23235663	28770975	27126581	25950424	30091146	22592671	20464762	17636827	27155888	18843630
23235621	28766313	27121755	25927089	24801225	22592704	20393958	17636790	14974041	23733365
23235665	28756618	27113258	25921416	27907224	22513919	20393959	25504256	28539008	15266523
23450556	28744896	27101360	25922859	24782322	22513931	20238347	24338345	14974076	29342498
30004571	28738443	27095103	25922858	24756895	22513977	20238359	17636756	22895917	24194466
29975402	28731502	27096326	25908299	24748537	22513971	29431872	17443591	22696338	24089293

29969521	28724193	27096438	25900537	24719028	22513901	20238341	29920656	14974022	26858174
29957861	28700811	27096578	25854213	24706397	22513964	20238338	17443530	14583927	25407135
29953167	28685503	27092951	25855461	24700291	22513963	20166059	17443505	14583945	27258214
29938790	28636204	27048768	25856365	24696265	22513932	20166067	19588449	14583969	30036453
29939406	28625021	30187450	25835053	24652601	27958643	20091625	17443627	27616189	30019767
29928755	28617932	27025289	25822346	24623260	22419339	20091599	17253577	26270620	29424930
29910691	28613416	27007217	25822171	24615288	22419289	20091665	17253467	14583967	28944453
29864216	28585288	26976671	25803542	24595586	26400848	20091498	22592707	14583926	28453187
29864793	28555461	26905373	25803793	24590623	22419320	20091546	28244064	14584019	28388808
29797578	28542712	26888026	25793972	24585221	22336858	20091505	17253544	28417451	28253424
29775501	28535332	26884379	25790326	24569952	22336798	30191554	23740669	12918001	27627458
29770432	28516471	26886473	25764172	24570033	22336866	20091540	17054141	29763508	22895947
29768662	28521070	26867004	29624206	24566946	22336792	20091659	17054175	12804463	22696347
29734473	28504837	26866512	25740785	29797697	22258983	25408622	17054261	12804441	24310847
29717488	28504325	26853855	25723574	22592719	22258943	25408623	17054137	12804462	24242360
29693726	28497473	26848926	25719222	29253322	22161389	21833935	17054183	12804452	20393943
29667726	28472862	26848807	25715323	24526330	28755534	25267900	17054173	17636748	18843705
29669176	28440853	26839116	28658720	24524153	22161416	29509957	17054277	12535487	29205264
29641848	28406263	26837472	25701429	24515444	22161372	19821323	27471845	12535504	27055154
29552749	28402017	26827159	25695214	24510679	22161379	26938118	17054269	12535399	16437457
29543326	28378502	26801659	25691262	24504983	22161390	19821399	17054211	12535466	16235308
29537066	28379619	26798057	25654768	24477672	22161392	19821310	16856018	12519621	25356786
29518253	28368091	26777994	25629415	24474579	22071846	19821359	16856014	12519595	17943772
29512818	28349529	26772902	25601826	24449085	22071835	19821267	16856110	12519591	11279769
29495063	28349512	26771164	25566754	24442580	22071802	19821384	16856077	29553158	20614462
29482264	28350426	26758576	25563827	24442763	22071816	19821364	16856089	12137677	21069689
29464690	28321847	26760424	25560834	24385423	22071840	19821369	16856075	28535578	11687182
29460276	28301050	26725721	25561409	24374605	26490698	19821380	16856104	29473717	

29446825	28290161	26711838	25549989	24362925	21975744	19821381	16856032	25739040	
29393511	28295158	26694853	25547090	24353997	21975780	19821362	16856102	12076408	
29363105	28295159	26690844	25536183	24347088	21975766	19821374	16625649	12076440	
29355908	28281295	26676202	25528992	24338106	21901707	19588359	16625563	12076483	
29338066	28244065	26666580	25523279	24323411	21901705	19588437	16625574	11869584	
29341071	28245343	26645724	25500895	24323843	21901734	25770311	16625528	11687074	
29320603	28245347	26646017	25493418	24307518	21901722	19588442	16625617	11687174	
29315455	28225198	26637264	25490118	24297506	21901694	22696319	16625644	11687148	
29304272	28178363	26630545	25486075	24277681	21901729	19588423	16625645	11686988	
29298459	28178770	26630252	25478936	24277645	21901715	19588413	16625607	11687147	
29297205	28155990	26606533	25431843	24265169	21901732	26436959	16625609	18254017	
29284075	28157274	26590358	25427719	24249541	21901675	19370667	16625543	11686955	
29278410	28125765	26576758	25425010	24249483	21901680	19370635	16437530	11405972	
29261853	28084646	26564018	25416857	24226506	21833969	19370584	16437442	11406054	
29238948	28066156	26558494	25410644	24222383	21833989	19370663	16437436	11279690	
29199769	28052316	26561745	25397904	24213953	21833960	25267894	16437473	11034691	
29192424	28002636	26558329	25372407	29534303	21833956	19160282	16437462	16437445	
29164603	27996087	26545176	25358850	24166676	21735405	19160300	16437523	11034672	
29130474	27995607	26523368	25352443	24163828	21735438	19160343	20556762	11034769	
29117629	27977844	26505729	25348770	24156097	21735413	19160339	21901693	11034754	
29099149	27933615	26457821	22895916	24151011	21735392	19160194	16235338	11034690	

text 1. Search terms for evaluating evidence of statistically significant results included in clinical management guidelines for 7 age-treatment subgroup analyses.

For the review by Rowe et al (Mar 2017):

- Google search terms: "Botulinum toxin for treating strabismus guideline recommendation"
- UpToDate:
    - https://www.uptodate.com/contents/evaluation-and-management-of-strabismus-in-children?topicRef=6255&source=see_link (last updated Sept 13, 2018; literature review current through Jun 2019)
    - https://www.uptodate.com/contents/overview-of-diplopia?search=strabismus&source=search_result&selectedTitle=3~150&usage_type=default&display_rank=3 (last updated Jan 16, 2019; literature review current through Jun 2019)
    - https://www.uptodate.com/contents/third-cranial-nerve-oculomotor-nerve-palsy-in-adults?search=strabismus&source=search_result&selectedTitle=2~150&usage_type=default&display_rank=2 (last updated Jun 19, 2017; literature review current through Jun 2019)
    - https://www.uptodate.com/contents/amblyopia-in-children-classification-screening-and-evaluation#H3058888 (last updated Sept 18, 2018; literature review current through Jun 2019)
- Cochrane Clinical Answers:
    - https://www.cochranelibrary.com/cca/doi/10.1002/cca.1687/full (published Jul 25, 2017)
- BMJ Best Practice:
    - https://bestpractice.bmj.com/topics/en-us/689 (last updated Nov 2017; last reviewed Jun 2019)

For the review by Kaner et al (Sep 2017):

- Google search terms: "Digital mobile interventions for reducing alcohol consumption guideline recommendation"
- UpToDate:
    - https://www.uptodate.com/contents/brief-intervention-for-unhealthy-alcohol-and-other-drug-use-efficacy-adverse-effects-and-administration#H2495523184 (last updated Jun 3, 2018; literature review current through Jun 2019)
    - https://www.uptodate.com/contents/psychosocial-treatment-of-alcohol-use-disorder?search=alcohol%20consumption%20intervention&source=search_result&selectedTitle=2~150&usage_type=default&display_rank=2 (last updated Sep 6, 2018; literature review current through Jun 2019)
    - https://www.uptodate.com/contents/approach-to-treating-alcohol-use-disorder?search=alcohol%20consumption%20intervention&source=search_result&selectedTitle=4~150&usage_type=default&display_rank=4 (last updated Nov 16, 2018; literature review current through Jun 2019)
- Cochrane Clinical Answers:
    - https://www.cochranelibrary.com/cca/doi/10.1002/cca.2086/full (published Jul 25, 2018)
    - https://www.cochranelibrary.com/cca/doi/10.1002/cca.1449/full (published Dec 28, 2016)
- BMJ Best Practice:

    - https://bestpractice.bmj.com/topics/en-us/198 (last updated Jun 2018; last reviewed Jun 2019)

For the review by Mhaskar et al (Oct 2014):

- Google search terms: “Colony-stimulating factors for chemotherapy-induced neutropenia guideline recommendation”
- UpToDate:
    - https://www.uptodate.com/contents/use-of-granulocyte-colony-stimulating-factors-in-adult-patients-with-chemotherapy-induced-neutropenia-and-conditions-other-than-acute-leukemia-myelodysplastic-syndrome-and-hematopoietic-cell-transplantation (last updated Nov 26, 2018; literature review current through Jun 2019)
    - https://www.uptodate.com/contents/fever-in-children-with-chemotherapy-induced-neutropenia/print (last updated Jul 3, 2018; literature review current through Jun 2019)
    - https://www.uptodate.com/contents/overview-of-neutropenic-fever-syndromes?topicRef=6051&source=see_link (last updated Nov 30, 2018; literature review current through Jun 2019)
    - https://www.uptodate.com/contents/diagnostic-approach-to-the-adult-cancer-patient-with-neutropenic-fever?topicRef=6051&source=see_link (last updated Dec 21, 2018; literature review current through Jun 2019)
- Cochrane Clinical Answers:
    - https://www.cochranelibrary.com/cca/doi/10.1002/cca.535/full (published Nov 25, 2014)
    - https://www.cochranelibrary.com/cca/doi/10.1002/cca.889/full (published Oct 6, 2015)
    - https://www.cochranelibrary.com/cca/doi/10.1002/cca.1743/full (published Aug 4, 2017)
    - https://www.cochranelibrary.com/cca/doi/10.1002/cca.1433/full (published Oct 12, 2016)
    - https://www.cochranelibrary.com/cca/doi/10.1002/cca.973/full (published Jul 11, 2016)
    - https://www.cochrane.org/CD003039/GYNAECA_does-administering-colony-stimulating-factors-plus-antibiotics-people-fever-and-low-white-cell-count (published Oct 30, 2014)
- BMJ Best Practice:
    - https://bestpractice.bmj.com/topics/en-us/950 (last updated Oct 2018; last reviewed Jun 2019)

For the review by Hemmingsen et al (Dec 2017):

- Google search terms: “Diet physical activity for incidence of type 2 diabetes guideline recommendation” & “Diet physical activity for two hour plasma glucose guideline recommendation”
- UpToDate:
    - https://www.uptodate.com/contents/epidemiology-presentation-and-diagnosis-of-type-2-diabetes-mellitus-in-children-and-adolescents (last updated Mar 2018; literature review current through Jun 2019)
    - https://www.uptodate.com/contents/risk-factors-for-type-2-diabetes-mellitus (last updated Oct 2, 2018; literature review current through Jun 2019)
    - https://www.uptodate.com/contents/prevention-of-type-2-diabetes-mellitus (last updated Jul 3, 2019; literature review current through Jun 2019)

- https://www.uptodate.com/contents/pathogenesis-of-type-2-diabetes-mellitus (last updated Nov 5, 2018; literature review current through Jun 2019)

- Cochrane Clinical Answers:
  - https://www.cochranelibrary.com/cca/doi/10.1002/cca.1859/full (published Nov 15, 2017)
  - https://www.cochranelibrary.com/cca/doi/10.1002/cca.1860/full (published Nov 15, 2017)
- BMJ Best Practice:
  - https://bestpractice.bmj.com/topics/en-gb/24 (last updated May 2019; last reviewed Jun 2019)

For the review by Sguassero et al (Jun 2012):

- Google search terms: "Supplementary feeding for weight gain children low middle income country guideline recommendation"
- UpToDate:
  - https://www.uptodate.com/contents/management-of-moderate-acute-malnutrition-in-children-in-resource-limited-countries (last updated Oct 19, 2018; literature review current through Jun 2019)
- Cochrane Clinical Answers:
  - https://www.cochranelibrary.com/cca/doi/10.1002/cca.2207/full (published Oct 10, 2018)
- BMJ Best Practice:
  - https://bestpractice.bmj.com/topics/en-us/1307 (last updated Mar, 2019; last reviewed Jun 2019)
  - https://bestpractice.bmj.com/topics/en-us/747 (last updated Jan 2018; last reviewed Jun 2019)

For the review by Adams et al (Oct 2007):

- Google search terms: "Fluticasone beclomethasone budesonide forced expiratory volume guideline recommendation"
- UpToDate:
  - https://www.uptodate.com/contents/asthma-treatment-in-adolescents-and-adults-beyond-the-basics (last updated Jan 8, 2019; literature review current through Jun 2019)
  - https://www.uptodate.com/contents/treatment-of-severe-asthma-in-adolescents-and-adults (last updated Mar 28, 2018; literature review current through Jun 2019)
  - https://www.uptodate.com/contents/asthma-in-children-younger-than-12-years-treatment-of-persistent-asthma-with-controller-medications (last updated Jan 8, 2018; literature review current through Jun 2019)
- Cochrane Clinical Answers:
  - https://www.cochranelibrary.com/cca/doi/10.1002/cca.349/full (published May 28, 2014)
  - https://www.cochranelibrary.com/cca/doi/10.1002/cca.350/full (published May 28, 2014)
- BMJ Best Practice:
  - https://bestpractice.bmj.com/topics/en-us/782 (last updated Jul 2018; last reviewed Jun 2019)
  - https://bestpractice.bmj.com/topics/en-us/44 (last updated Jun 2018; last reviewed Jun 2019)

**table S2**: Reasons for not performing age-treatment subgroup analyses among 162 Cochrane intervention reviews.	
**Reason**	**No. of Articles (%)**
No eligible studies found using inclusion criteria; therefore, subgroup analyses not possible	16 (9.9)
Eligible studies found but no meta-analyses conducted	22 (13.6)
Insufficient data identified for subgroup analyses	71 (43.6)
Identified studies were not able to be pooled in a way that allowed for subgroup analyses	8 (4.9)
Pre-specified some criteria for heterogeneity level required to perform analyses, and that threshold was not met	5 (3.1)
Stated that age would be a factor considered for exploration if heterogeneity was identified	13 (8.0)
No statement given, and the reason was not clearly inferable from the text	20 (12.4)
Age-treatment analyses appeared to be performed in the text but not reported in forest plots	2 (1.2)
Stated only one age-treatment subgroup level to identify without explicitly stating the comparison group	3 (1.9)
Other reason	2 (1.2)

**table S3**: Characteristics of 97 age-treatment subgroup analyses from 25 CDSR reviews.							
**PubMed Identifier (Year)**	**Indication**	**Population Characteristics**	**Comparison**	**Outcome**	**Subgroup Levels**	**No. of Unique Trials Included**	**Reported *P* value?**
11279769 (2001)	Respiratory	Children (>2 years) and adults with a clinical diagnosis of asthma, only including participants with both chronic obstructive pulmonary disease and asthma if data for asthma were reported separately	Beclomethasone dipropionate vs beclomethasone dipropionate: Parallel design, no oral steroids, 400 mcg/d v 800 mcg/d	Change in Morning peak expiratory flow rate (liters/min) compared to baseline (2.4)	Children; Adults	2	Y
				Change in Evening peak expiratory flow rate (liters/min) compared to baseline (2.5)	Children; Adults	2	Y
			Beclomethasone dipropionate vs beclomethasone dipropionate: Crossover design, no oral steroids, 400 mcg/d v 800 mcg/d	Forced expiratory volume 1 (liters) (5.1)	Children; Adults	2	Y
				Morning peak expiratory flow rate (liters/min) (5.3)	Children; Adults	2	Y
				Evening peak expiratory flow rate (liters/min) (5.4)	Children; Adults	2	Y
11687182 (2001)	Respiratory	Children (>2 years) and adults with chronic asthma, only including participants with both chronic obstructive pulmonary disease and asthma if data for asthma were reported separately	Budesonide vs budesonide: Parallel design, not on oral steroids: 400 v 800 mcg/d	Withdrawal due to asthma exacerbation (No. of patients) (3.12)	Children; Adults	2	N
			Budesonide vs budesonide: Parallel design, not on oral steroids: 200 v 800 mcg/d	Withdrawal due to asthma exacerbation (No. of patients) (5.9)	Children; Adults	4	N
			Budesonide vs budesonide: Parallel	Withdrawal due to asthma exacerbation	Children; Adults	2	N

			design, oral steroid treated: 200 v 400 mcg/d	(No. of patients) (11.1)			
			Budesonide vs budesonide: Parallel design, oral steroid treated: 400 v 800 mcg/d	Withdrawal due to asthma exacerbation (No. of patients) (12.1)	Children; Adults	2	N
			Budesonide vs budesonide: Parallel design, oral steroid treated: 200 v 800 mcg/d	Withdrawal due to asthma exacerbation (No. of patients) (13.1)	Children; Adults	2	N
16235308 (2005)	Infectious	Healthy children 0 - 18 years of age with chickenpox	Acyclovir versus placebo	Time to no new lesions (1.1)	Ages 2 - 12 years; 5 - 16 years; 13 - 18 years	3	N
				Maximum number of new lesions (1.2)	Ages 2 - 12 years; 5 - 16 years; 13 - 18 years	3	N
				Time to no fever (1.3)	Ages 2 - 12 years; 5 - 16 years; 13 - 18 years	3	N
				Time to no itching (1.4)	Ages 2 - 12 years; 5 - 16 years	2	N
				Skin complications (1.5)	Ages 2 - 12 years; 5 - 16 years; 13 - 18 years	3	N
				Central nervous system complications (1.6)	Ages 2 - 12 years; 5 - 16 years	2	N
				Respiratory complications (1.7)	Ages 2 - 12 years; 5 - 16 years	2	N
16437457 (2006)	Infectious	Children (<15 years) who are affected with HIV, or less than 18 months with HIV-infected mothers	Cotrimoxazole versus control	Death by age (1.4)	Ages < 2 years; > 2 years	1	N
17943772 (2007)	Respiratory	Children (>2 years) and adults with chronic asthma, only including participants with both	Fluticasone propionate versus beclomethasone dipropionate or	Forced expiratory volume 1 (5.1)	Children; Adults	17	Y
				Change in Forced expiratory volume 1	Children; Adults	12	Y

		COPD and asthma if data for asthma were reported separately	budesonide, parallel group studies: dose ratio 1:2	compared to baseline (5.2)			
				Forced expiratory volume 1 predicted (5.3)	Children; Adults	7	Y
				Change in forced expiratory volume 1 predicted (5.4)	Children; Adults	6	Y
				Forced vital capacity (5.5)	Children; Adults	9	Y
				Mean morning peak expiratory flow rate (5.7)	Children; Adults	12	Y
				Mean change in morning peak expiratory flow rate (5.8)	Children; Adults	17	Y
				Mean evening peak expiratory flow rate (5.9)	Children; Adults	10	Y
				Change in evening peak expiratory flow rate compared to baseline (5.10)	Children; Adults	10	Y
				Clinic peak expiratory flow (5.11)	Children; Adults	12	Y
				Change in Clinic peak expiratory flow rate (5.12)	Children; Adults	6	Y
			Fluticasone propionate versus beclomethasone dipropionate or budesonide, parallel	Forced expiratory volume 1 (6.1)	Children; Adults	10	Y
				Change in forced expiratory volume 1 (6.2)	Children; Adults	5	Y

			group studies: dose ratio 1:1	Forced expiratory volume 1 predicted (6.3)	Children; Adults	4	Y
				Forced vital capacity (6.4)	Children; Adults	6	Y
				Morning peak expiratory flow rate (6.5)	Children; Adults; Unclear	11	Y
				Change in morning peak expiratory flow rate compared to baseline (6.6)	Children; Adults	5	Y
				Morning peak expiratory flow rate predicted (6.7)	Children; Adults	3	Y
				Evening peak expiratory flow rate (6.8)	Children; Adults; Unclear	10	Y
				Change in evening peak expiratory flow compared to baseline (6.9)	Children; Adults	4	Y
				Clinic peak expiratory flow rate (6.11)	Children; Adults	9	Y
				Clinic peak expiratory predicted (6.12)	Children; Adults	4	Y
18843705 (2008)	Dermatology	Individuals of any age or gender with eczema diagnosed by a doctor, excluding other specific forms of eczema such as contact eczema	Probiotic versus placebo	Participant/parent-rated global change in symptoms of eczema at the end of treatment (1.15)	Ages < 2 years; 2 - 12 years; Not categorized	12	N
				Participant/parent-rated symptoms of eczema (Scoring of	Ages < 2 years; 2 - 12 years; Not categorized; Adults only	3	N

				atopic dermatitis part C) at the end of treatment (1.16)			
				Global eczema severity score (Total Scoring of atopic dermatitis) at the end of treatment (1.17)	Ages < 2 years; 2 - 12 years; Not categorized; Adults only	24	N
20393943 (2010)	Respiratory	Children (>2 years), adolescents, and adults with recurrent or chronic asthma	Long-acting beta2-agonist + inhaled corticosteroids versus higher dose inhaled corticosteroids	No. of patients with exacerbations requiring oral steroids (2.1)	Children; Adults	25	N
				No. of patients with exacerbations requiring hospitalization (2.12)	Children; Adults; Children and adults	33	N
20614462 (2010)	Gastrointestinal	Children and adults diagnosed with chronic constipation (Rome III criteria) or fecal impaction treated with lactulose or polyethylene glycol	Stool frequency per week	Stool frequency per week (1.3 & 1.4)	Children; Adults	5	N
			Form of stool	Form of stool (2.3 & 2.4)	Children; Adults	2	N
			Relief of abdominal pain	Relief of abdominal pain (3.2 & 3.3)	Children; Adults	3	N
			Did not require additional products	Did not require additional products (4.2 & 4.3)	Children; Adults	3	N
21069689 (2010)	Neoplastic	Men with confirmed prostate cancer (verified by cytological or histological examination), which is believed to be still confined to the prostate gland	Radical prostatectomy (RP) versus watchful waiting (WW)	Overall mortality - SPCG-4 Trial (1.2)	Ages < 65 years; ≥ 65 years	1	N
				Mortality due to prostate cancer - 12 year follow up (1.4)	Ages < 65 years; ≥ 65 years	1	N
				Distant metastases (1.5)	Ages < 65 years; ≥ 65 years	1	N

22696347 (2012)	Dietary	Children from low and middle income countries born at term (≥ 37 completed weeks of gestation), from birth to five years old, excluding children with malnutrition not resulting from insufficient dietary intake	Supplementary feeding	Weight (kg) gain during the intervention (12.3)	Children younger than 24 months; older than 24 months	5	Y
				Length/height (cm) gain during the intervention (12.4)	Children younger than 24 months; older than 24 months	5	Y
				Weight-for-length/height z-score at the end of the intervention (12.7)	Children younger than 24 months; other age range (6 months to 6 years)	3	Y
22895947 (2012)	Neoplastic	Women with advanced epithelial ovarian cancer (stage III/IV, as defined by the Federation of International Gynecologists and Obstetricians)	Neoadjuvant chemotherapy versus primary debulking surgery	Overall survival (1.2)	Ages < 50 years; 50 - 70 years; > 70 years	1	Y
24242360 (2013)	Psychiatric	Any individual with schizophrenia or similar serious, non-affective psychosis diagnosed by any criteria, and any trial where the majority of participants suffered from serious functional psychotic illness, such as schizophrenia	Haloperidol versus placebo	Global state: Overall improvement: No marked global improvement, > 6-24 weeks (clinician rated) (1.19)	Ages < 18 years; 18 - 65 years	8	Y
24310847 (2013)	Dental	Children (<16 years) or adults (>16 years), excluding participants with periodontitis at baseline, patients selected due to a pre-existing health condition, and studies where the majority of participants had orthodontic appliances, and those taking another prophylactic regimen for plaque/gingivitis unless separate data for triclosan/copolymer/fluoride	Caries	Caries increment at 30 to 36 months (DFT) (4.1)	Children (1100 ppm F, 0.243% NaF); Adults (1100 ppm F, 0.243% NaF); Adults (1500 ppm F, 0.331% NaF)	3	Y
				Caries increment at 24 to 36 months (DFS) (4.2)	Children (1100 ppm F, 0.243% NaF); Adults (1100 ppm F, 0.243% NaF); Adults (1500 ppm F, 0.331% NaF)	4	Y

		and fluoride arms were reported separately					
25356786 (2014)	Infectious	Individuals undergoing chemotherapy for cancer who experienced neutropenia (absolute neutrophil count < $1x10^9$/L (1000/mm^3)) and fever (body temperature higher than 38.5 degrees Celsius on one occasion or higher than 38 degrees Celsius on two or more occasions)	Colony-stimulating factor plus antibiotics versus antibiotics alone	Patients with hospitalizations for greater than 10 days (2.1)	Children; Adults	7	Y
				Time to neutrophil recovery (2.2)	Children; Adults	5	Y
				Duration of grade IV neutropenia (2.3)	Children; Adults	10	Y
				Time to recovering from fever (2.4)	Children; Adults; Children and adults	10	Y
27055154 (2016)	Respiratory	Children and adults with defined cystic fibrosis diagnosed clinically and by quantitative sweat chloride testing or genetic testing or both, including people with cystic fibrosis at all stages of lung disease	Oral nonsteroidal anti-inflammatory drug versus placebo	Annual rate of change in % predicted forced expiratory volume 1 (1.2)	Ages < 13 years at randomization; ≥ 13 years at randomization	2	Y
				Annual rate of change in % predicted forced vital capacity (1.4)	Ages < 13 years at randomization; ≥ 13 years at randomization	2	Y
				Annual rate of change in % predicted forced expiratory flow 25-75% (1.6)	Ages < 13 years at randomization; ≥ 13 years at randomization	2	Y
				Annual rate of change in % ideal body weight (1.11)	Ages < 13 years at randomization; ≥ 13 years at randomization	1	Y
				Chest X-ray score (1.13)	Ages < 13 years at randomization; ≥ 13 years at randomization	1	Y
27258214 (2016)	Cardiovascular	Individuals (>40 years), including patients with implantable pacemakers or defibrillators or a previous	Screening versus routine practice	Detection of new cases of atrial fibrillation (systematic) (1.4)	Ages 65-74 years; > 74 years	1	Y

		diagnosis of atrial fibrillation as long as these patients were excluded		Detection of new cases of atrial fibrillation (opportunistic) (1.6)	Ages 65-74 years; > 74 years	1	Y
			Systematic versus opportunistic screening	Age subgroups (2.3)	Ages 65-74 years; > 74 years	1	Y
27627458 (2016)	Procedural	Infants and adolescents (>1 month, <18 years) undergoing arterial line placement, excluding neonates	Ultrasound-guided arterial cannulation versus other techniques (palpation/Doppler)	Successful cannulation at the first attempt (1.2)	Infants and small children; Older children	4	Y
28253424 (2017)	Ophthalmolo gical	Children and adults with strabismus suitable for treatment with botulinum toxin to align the angle of deviation	Botulinum toxin versus surgery	Primary outcome - improved ocular alignment ≤ 10 prism dioptres (1.1)	Children; Adults	3	Y
28388808 (2017)	Procedural	All participants who have undergone lumbar puncture for medical reasons	Larger gauge traumatic needles versus smaller gauge traumatic needles	Post-dural puncture headache (2.3)	No distinctions about age; children; ages > 60 years	10	N
28453187 (2017)	Cardiovascul ar	Adults (>18 years) with or without a prior history of cardiovascular disease, including participants with normal lipid levels or hypercholesterolemia	Proprotein convertase subtillisin/kexin type 9 inhibitors versus placebo	Mean percentage change in low density lipoprotein cholesterol (9)	Ages < 65 years; ≥ 65 years	1	Y
			Proprotein convertase subtillisin/kexin type 9 inhibitors versus ezetimibe	Mean percentage change in low density lipoprotein cholesterol (10)	Ages < 65 years; ≥ 65 years	1	Y
28944453 (2017)	Behavioral	Only people in the community whose alcohol consumption had been screened as hazardous or harmful and were directed toward any digital intervention	Digital intervention versus no or minimal intervention	Quantity of drinking (grams/week), based on longest follow-up (1.3)	Adolescents/young adults; Adults	42	Y
29205264 (2017)	Endocrinal	Individuals diagnosed with intermediate hyperglycemia or		All-cause mortality (2.4)	Ages < 50 years; ≥ 50 years	10	Y

		prediabetes at increased risk of developing type II diabetes mellitus	Diet plus physical activity versus comparator	Incidence of type 2 diabetes (2.11)	Ages < 50 years; ≥ 50 years	11	Y
				Fasting plasma glucose (2.21)	Ages < 50 years; ≥ 50 years	10	Y
				2 hour plasma glucose (2.26)	Ages < 50 years; ≥ 50 years	9	Y
29424930 (2018)	Respiratory	Children (<18 years) or adults (>18 years) with cystic fibrosis diagnosed clinically and by sweat or genetic testing)	Mannitol versus control - cross-over studies of individuals with cystic fibrosis	Forced expiratory volume 1 % predicted (absolute change from baseline) (2.2)	Ages 6 - 11 years; 12 - 17 years	1	Y
30019767 (2018)	Cardiovascular	Adults (>18 years), excluding participants who were pregnant or acutely ill (with current cancer and undergoing heart or renal transplantation, with HIV or AIDS, on hemodialysis, with immunoglobulin A glomerulonephritis, or another renal problem except diabetic nephropathy) and trials with less that 90% of participants aged > 18 years or where adult data could not be separated from non-adult data	Higher polyunsaturated fatty acids (PUFA) versus lower PUFA intake - primary outcomes	All-cause mortality (1.10)	Mean age < 50 years; 50 to < 65 years; 65+ years	24	Y
				Coronary heart disease events (1.22)	Mean age < 50 years; 50 to < 65 years; 65+ years; Unclear	15	Y
				Stroke (1.34)	Mean age < 50 years; 50 to < 65 years; 65+ years; Unclear	11	Y
				Major adverse cardiac & cerebrovascular events (1.48)	Mean age < 50 years; 50 to < 65 years; 65+ years	2	Y
			Higher polyunsaturated fatty acids (PUFA) versus lower PUFA intake - dichotomous secondary outcomes	Cardiovascular mortality (2.10)	Mean age < 50 years; 50 to < 65 years; 65+ years; Unclear	16	Y
				Cardiovascular disease events (2.22)	Mean age < 50 years; 50 to < 65 years; 65+ years; Unclear	21	Y
				Coronary heart disease mortality (2.34)	Mean age < 50 years; 50 to < 65 years; 65+ years	10	Y
				Atrial fibrillation (2.48)	Mean age < 50 years; 50 to < 65 years; 65+ years; Unclear	10	Y

			Higher polyunsaturated fatty acids (PUFA) versus lower PUFA intake - continuous secondary outcomes	Body weight, kg (3.10)	Mean age < 50 years; 50 to < 65 years; 65+ years	15	Y
				BMI, kg/m^2 (3.22)	Mean age < 50 years; 50 to < 65 years; 65+ years	11	Y
				Total cholesterol, mmoL/L (3.37)	Mean age < 50 years; 50 to < 65 years; 65+ years; Unclear	29	Y
				Total glycerides, mmoL/L (3.49)	Mean age < 50 years; 50 to < 65 years; 65+ years; Unclear	22	Y
				High density lipoprotein, mmoL/L (3.61)	Mean age < 50 years; 50 to < 65 years; 65+ years; Unclear	20	Y
				Low density lipoprotein, mmoL/L (3.73)	Mean age < 50 years; 50 to < 65 years; 65+ years; Unclear	17	Y
30036453 (2018)	Neoplastic	Participants with locally advanced or metastatic urothelial carcinoma of the bladder as determined by imaging or biopsy, whose disease progressed during or following platinum-containing chemotherapy, excluding participants receiving pembrolizumab as first-line therapy	Pembrolizumab versus chemotherapy (post-hoc)	Overall survival based on age (3.1)	Ages < 65 years; ≥ 65 years	1	Y

**table S4**: Meta-analytical methods used by authors in their age-treatment interactions.		
**Method Used**	**Subgroup analyses with non-overlapping subgroup levels**	**Subgroup analyses with overlapping subgroup levels**
All age-treatment analyses	65	32
Effect Measure		
Mean Difference	40	17
Risk Ratio	15	13
Statistical Method		
Inverse Variance	46	19
Mantel-Haenszel	18	13
Analysis Model		
Fixed	52	6
Random	13	26

**table S5. Summary results for proportion of statistically significant age-treatment interactions based on different characteristics and criteria among subgroup analyses with non-overlapping subgroup levels.**									
	**No (%) of statistically significant age-treatment interactions**								
	P value for interaction reported in forest plots			*P* value for interaction not reported in the forest plots			Total		
		P < 0.05	*P* < 0.005		*P* < 0.05	*P* < 0.005		*P* < 0.05	*P* < 0.005
All age-treatment analyses									
Using analytical methods reported in forest plots[a]	n = 51	7 (13.7)	2 (3.9)	n = 14	4 (28.6)	1 (7.1)	n = 65	11 (16.9)	3 (4.6)
Standardized using a fixed effects model[b]	n = 49	8 (16.3)	2 (4.1)	n = 14	5 (35.7)	2 (14.3)	n = 63	13 (20.6)	4 (6.3)
Standardized using a random effects model[b]	n = 49	7 (14.3)	2 (4.1)	n = 14	4 (28.6)	1 (7.1)	n = 63	11 (17.5)	3 (4.8)
One age-treatment analysis per treatment comparison[c]									
Using analytical methods reported in forest plots[a]	n = 25	5 (20.0)	1 (4.0)	n = 13	4 (30.8)	1 (7.7)	n = 38	9 (23.7)	2 (5.3)
Standardized using a fixed effects model[b]	n = 23	6 (26.1)	1 (4.3)	n = 13	5 (38.5)	2 (15.4)	n = 36	11 (30.6)	3 (8.3)
Standardized using a random effects model[b]	n = 23	5 (21.7)	1 (4.3)	n = 13	4 (30.8)	1 (7.7)	n = 36	9 (25.0)	2 (5.6)
One age-treatment analysis per review[c]									
Using analytical methods reported in forest plots[a]	n = 15	4 (26.7)	2 (13.3)	n = 5	2 (40.0)	0 (0.0)	n = 20	6 (30.0)	2 (10.0)
Standardized using a fixed effects model[b]	n = 14	5 (35.7)	1 (7.1)	n = 5	2 (40.0)	1 (20.0)	n = 19	7 (36.8)	2 (10.5)
Standardized using a random effects model[b]	n = 14	4 (28.6)	1 (7.1)	n = 5	2 (40.0)	0 (0.0)	n = 19	6 (31.6)	1 (5.3)

[a] We recreated the forest plots using the same methods outlined in the original Cochrane review (i.e., if the authors applied the Dersimonian & Laird random effects model to summarize risk ratios, we use the same effect measure and model).
[b] When standardizing using fixed and random effects models, we excluded two subgroup analyses from one Cochrane review that did not provide information on which studies were included in the subgroup analyses or the methodology for the subgroup analyses that they conducted.
[c] Using the primary outcome described in the text, if available, and otherwise using the outcome with the most data included (number of trials, or, in the event of a tie, the smallest variance in the summary effect).

**text 2**. Standardization using only fixed and only random effects models.
After excluding two subgroup analyses that did not provide information on which studies were included in the subgroup analyses and standardizing the calculations using only fixed and only random effects (Dersimonian and Laird) models with standard effect measures (risk ratio or mean difference), eight (8 of 49, 16.3%) and seven (7 of 49, 14.3%) of the 49 analyses were statistically significant, respectively. Among the 14 analyses that did not report a *P* value from an interaction test, four (28.6%) were statistically significant using the authors' outlined methods and a random effects model, and five using a fixed effects model.<br><br>In both sensitivity analyses, after excluding the two subgroup analyses that did not provide information regarding the individual studies included in the analyses, standardization using the random effects model did not change the number of analyses with statistically significant age-treatment interactions (9 of 36, 25.0%; 6 of 19, 31.6%) (**Additional file 1: Supplementary Table 5**). Standardization using a fixed effects model resulted in 11 (11 of 36, 30.6%) and seven (7 of 19, 36.8%) analyses with a statistically significant *P* value from an interaction test when selecting one analysis per treatment comparison and one analysis per Cochrane review, respectively (**Additional file 1: Supplementary Table 5**).
